# Use-case scenarios for an anti-*Cryptosporidium* therapeutic

**DOI:** 10.1371/journal.pntd.0009057

**Published:** 2021-03-11

**Authors:** Paul G. Ashigbie, Susan Shepherd, Kevin L. Steiner, Beatrice Amadi, Natasha Aziz, Ujjini H. Manjunatha, Jonathan M. Spector, Thierry T. Diagana, Paul Kelly

**Affiliations:** 1 Novartis Institute for Tropical Diseases, Emeryville, California, United States of America; 2 Alliance for International Medical Action (ALIMA), Dakar, Senegal; 3 The Ohio State University, Columbus, Ohio, United States of America; 4 Children’s Hospital, University Teaching Hospitals, Lusaka, Zambia; 5 Tropical Gastroenterology & Nutrition Group, University of Zambia School of Medicine, Lusaka, Zambia; 6 Blizard Institute, Barts & The London School of Medicine, Queen Mary University of London, London, United Kingdom; McGill University, CANADA

## Abstract

*Cryptosporidium* is a widely distributed enteric parasite that has an increasingly appreciated pathogenic role, particularly in pediatric diarrhea. While cryptosporidiosis has likely affected humanity for millennia, its recent “emergence” is largely the result of discoveries made through major epidemiologic studies in the past decade. There is no vaccine, and the only approved medicine, nitazoxanide, has been shown to have efficacy limitations in several patient groups known to be at elevated risk of disease. In order to help frontline health workers, policymakers, and other stakeholders translate our current understanding of cryptosporidiosis into actionable guidance to address the disease, we sought to assess salient issues relating to clinical management of cryptosporidiosis drawing from a review of the literature and our own field-based practice. This exercise is meant to help inform health system strategies for improving access to current treatments, to highlight recent achievements and outstanding knowledge and clinical practice gaps, and to help guide research activities for new anti-*Cryptosporidium* therapies.

## Introduction

Diarrhea has long been one of humanity’s gravest health problems. In 2017, there were greater than 6 billion incident episodes and 1.5 million deaths from diarrhea, making it the fifth leading cause of years of life lost [1,2]. The clinical impact is particularly severe in children under age 5, in whom diarrhea is the third most common cause of mortality (behind pneumonia and preterm birth complications) with nearly half a million attributable deaths each year [3,4]. Fortunately, mortality due to diarrhea has decreased, from more than 2.5 million overall deaths and 1.6 million child deaths in 1990, thanks in part to oral rehydration therapy and various public health advances including rotavirus vaccine and improved water and sanitation [2,5,6]. While assuring universal access to these proven interventions remains an urgent priority, there is also a need to identify new paths to tackling specific aspects of diarrheal disease in order to maintain, or ideally accelerate, progress.

Over the past decade, a number of large, multicountry studies have cast light on the global epidemiology of pediatric diarrhea in the modern era. A persistent area of uncertainty in management of diarrhea relates to appropriate use of targeted therapies. Dozens of enteric organisms (including bacteria, viruses, and protozoa) induce diarrhea and, while most episodes are self-limiting or can be successfully managed with supportive care, there are recognized clinical scenarios where pathogen-specific treatment is indicated (Table 1). If novel data demonstrate that certain additional patient populations may benefit from targeted treatment approaches, then translating evidence into clear and actionable guidance for frontline health workers provides an opportunity to further reduce preventable diarrhea-associated harm.

**Table 1:** Clinical scenarios where pathogen- or syndrome-specific treatment is applied for diarrheal disease. This table summarizes relevant aspects of treatment guidelines published by the World Health Organization and other groups. It is acknowledged that the evidence base for some interventions is variable and the use of these therapies in practice is often country- and resource-dependent.

Disease	Clinical scenario	Typical therapeutic interventions
Amoebiasis	• Bloody diarrhea in severely malnourished children with *Entamoeba histolytica* or bloody diarrhea that continues after treatment for *Shigella* [13,14] • Patients with trophozoites of *E*. *histolytica* containing red blood cells identified in fresh feces [15]	Metronidazole, tinidazole
Cholera	• Adults and children over 5 years with severe dehydration from acute watery diarrhea (usually with vomiting) [14,15] • Children greater than 2 years with acute watery diarrhea in an area with cholera outbreak [14,15]	Co-trimoxazole, doxycycline, erythromycin, fluoroquinolones, tetracycline
*Clostridium difficile* infection	Hospital- or community-acquired diarrheal disease [16]	Oral vancomycin, metronidazole
Giardiasis	• Severely malnourished children with *Giardia* [13,17] • Children with chronic, malabsorptive, nonbloody diarrhea without fever • Adults and children with stool microscopy that is positive for cysts or trophozoites [14]	Metronidazole, tinidazole, ornidazole, nitaxozanide
Shigellosis	Bloody diarrhea in severely malnourished children [13,18]	Ampicillin, cotrimoxazole, nalidixic acid
	Bloody diarrhea in adults and children, irrespective of nutritional status [14,18]	Azithromycin, ceftriaxone, ciprofloxacin
Other clinical scenarios	Bloody diarrhea in HIV–positive and HIV–exposed infants and children [19]	Ciprofloxacin
	Diarrhea in returned travelers to low-income countries [20]	Ciprofloxacin, azithromycin

*Cryptosporidium* is a widely distributed enteric parasite that has an increasingly appreciated pathogenic role, particularly in pediatric diarrhea. In the 1980s, cryptosporidiosis was recognized to be a serious enteric infection in people living with HIV/AIDS (PLWHA) and indeed was one of the original acquired immunodeficiency syndrome (AIDS)-defining illnesses [7,8]. Since that time, *Cryptosporidium* has been implicated in acute or chronic diarrhea in varied patient populations and in diverse geographies, especially among children in low- and middle-income countries (LMICs) [9].

There is no vaccine for cryptosporidiosis, and the single approved medicine, nitazoxanide, appears to be infrequently used, presumably due to a mix of factors including perception of limited efficacy (small-scale clinical studies in sub-Saharan Africa demonstrated poor responses in malnourished children with HIV infection) [10,11], diagnostic challenges (resources are largely absent in clinical settings in LMICs to identify *Cryptosporidium* oocysts by microscopy or point-of-care rapid diagnostic tests), and dearth of guidance for addressing cryptosporidiosis in standard treatment guidelines published by international and national authorities [10–12]. Given that cryptosporidiosis is rarely specifically treated, practitioners in regions where it is highly prevalent may naturally have limited awareness of *Cryptosporidium* as a potential pathogen and restricted understanding of when anti-*Cryptosporidium* therapy may have clinical benefit.

As a group of physicians and scientists engaged on the problem of diarrheal diseases in LMICs, along with researchers conducting drug discovery for new anti-*Cryptosporidium* medicines, we sought to plainly characterize salient issues relating to therapies for cryptosporidiosis. Specifically, we compiled from contemporary literature and from firsthand experience a description of the populations most affected by cryptosporidiosis. We then reviewed the relevance of nitazoxanide in those patients and examined the clinical settings in which an anti-*Cryptosporidium* medicine is needed. Finally, we summarized the expected characteristics and benefits of an ideal therapeutic and assessed the landscape of anti-*Cryptosporidium* therapies in the drug development pipeline. This exercise is meant to help inform health system strategies for improving access to nitazoxanide in clinical circumstances where it might be indicated, to highlight key knowledge and clinical practice gaps relating to *Cryptosporidium’s* influence on human health, and to help guide research activities for new anti-*Cryptosporidium* therapies.

## A brief history of cryptosporidiosis

*Cryptosporidium* species are ubiquitous Apicomplexan protozoa phylogenetically related to *Plasmodium* (the agent of malaria), which are thought to have evolved into existence on the order of 600 million years ago [21]. However, while malaria has long been recognized to be an ancient scourge, the impact of *Cryptosporidium* on human health has only been appreciated in recent history. *Cryptosporidium* was first observed in mice in 1907, and its presence as a pathogen in young cows was described in 1971 [22,23]. The first human infection was reported in 1975 (a 3-year-old farm girl from rural Tennessee with severe diarrhea, vomiting, and abdominal pain) [24]. Shortly thereafter, as the HIV/AIDS epidemic surfaced, *Cryptosporidium* was associated with severe, sometimes fatal diarrhea in immunocompromised individuals [25,26]. The first reports of cryptosporidiosis in Africa came from Liberia and Rwanda beginning in 1984 [27,28], and since then, it has been described on every continent (including Antarctica) in both low- and high-resource countries.

More than 15 species of *Cryptosporidium* cause human infection with 2 species accounting for approximately 90% of human infections: *Cryptosporidium hominis* (approximately 80%) and *Cryptosporidium parvum* (approximately 10%) [29]. *Cryptosporidium* is hardy. The oocysts can survive in the environment for months and are resistant to chlorine concentrations that are normally used for water treatment [30,31]. Transmission between humans takes place via waterborne, foodborne, and nosocomial routes; there is also anthroponotic and zoonotic spread. As few as 10 oocysts can cause clinically apparent infection in healthy adults [32]. The pathogenicity of *Cryptosporidium* seems to be influenced by the parasite species and the type, age, and immune status of the infected host [33]. As described in greater detail in the sections below, it is increasingly suspected that both symptomatic as well as asymptomatic infections have negative implications on human health.

Looking ahead, there are signs that the burden of cryptosporidiosis globally could rise even more due to changes in climate, urbanization (facilitating increases in person-to-person transmission), and predicted population growth patterns. Warmer temperatures and changing rainfall patterns may cause increased contamination of water bodies, greater transmission, and emergence of disease in new geographies [34]. Modeling exercises show *Cryptosporidium* emission to the environment could increase up to 70% by the year 2050 in some regions [35]. Moreover, impacts of climate change on food security and nutritional status may further increase susceptibility of vulnerable populations, especially children, to cryptosporidiosis and other parasitic infections [36,37].

## Patient groups in need of an anti-*Cryptosporidium* therapeutic

There is currently compelling evidence of unmet therapeutic need for enteric cryptosporidiosis in 3 patient groups specifically: young children aged 0 to 24 months in LMICs, malnourished children under age 5, and immunosuppressed individuals of any age. These patient groups appear to be at elevated risk of acquiring *Cryptosporidium* infection, and, when infected, studies have shown patients in these populations to have poorer clinical outcomes. In addition, people in any global setting that are affected by outbreaks may be candidates for *Cryptosporidium*-specific therapy. A brief review of the literature pertaining to these patient groups is presented below and summarized in Table 2. Also discussed are the theoretical cases for empiric therapy and mass drug administration.

**Table 2:** Summary of use-case scenarios for an anti-*Cryptosporidium* therapeutic.

Use-case	Disease burden	Potential treatment sites	Potential treatment strategies	Current applicability of nitazoxanide
Young children aged 0–24 months	7.5 million cases and 200,000 deaths annually (in Africa and Asia) [29]	Primary, secondary, and tertiary health facilities in LMICs	Diagnosis-based treatment	Not approved in children under 12 months
			Empiric treatment in high-risk populations where diagnostic tools are not available	Insufficient evidence and guidelines
		Community-based treatment	Mass drug administration in seasons with high prevalence	Insufficient evidence and guidelines
Malnourished children	Estimated 50 million wasted children globally [96]; recent studies indicate 10%–20% prevalence of cryptosporidiosis in children with acute malnutrition [54–56]	Primary, secondary, and tertiary health facilities in LMICs; malnutrition care centers in clinics and hospitals	Diagnosis-based treatment (however, diagnostic challenges in typical clinical settings often limit practicality of this approach)	Poorly effective (i.e., less than 50% efficacious) [10]
			Empiric treatment in high-risk populations where diagnostic tools are not available	Insufficient evidence and guidelines; nitazoxanide poorly effective [10]
Immunocompromised persons	Estimates range from 5%–50% of PLWHA and up to 30% of solid organ transplant recipients [80,84,97,98]	Primary, secondary, and tertiary health facilities in LMICs; HIV/AIDS treatment programs; transplant centers in any global setting	Diagnosis-based treatment	Poorly or noneffective for PLWHA [10,11,99]
			Empiric treatment in high-risk populations where diagnostic tools are not available	Insufficient evidence and guidelines; nitazoxanide poorly effective for PLWHA [10]
Outbreaks	Hundreds of outbreaks reported annually in high-income countries that likely affect thousands of individuals [100,101]; poor data availability for outbreak reporting in LMICs	Health facilities involved in outbreak response	Diagnosis-based treatment	Not approved in children under 12 months
			Empiric treatment where diagnostic tools are not available	Insufficient evidence and guidelines
		Community-based treatment	Mass drug administration during outbreaks	Insufficient evidence and guidelines

LMICs, low- and middle-income countries; PLWHA, people living with HIV/AIDS.

### Young children aged 0 to 24 months in LMICs

In the past decade, *Cryptosporidium* has emerged to be one of the most important diarrhea-causing enteric pathogens associated with severe morbidity and mortality in immunocompetent young children under 2 years of age. The strongest evidence comes from 2 major prospective studies, the Global Enteric Multicenter Study (GEMS) [38] and the Etiology, Risk Factors, and Interactions of Enteric Infections and Malnutrition and the Consequences for Child Health and Development Project (MAL-ED) [39]. In addition, various sub-analyses and meta-analyses utilizing data from those studies have further reported on the burden of cryptosporidiosis. The findings from GEMS and MAL-ED are also consistent with numerous recent reports from smaller scale investigations in Africa and Asia [40,41].

The original GEMS study (GEMS-1), which took place from 2007 to 2011, involved nearly 10,000 children aged 0 to 59 months who presented at health facilities with moderate-to-severe diarrhea (MSD) and more than 13,000 control children without diarrhea at 4 sites in Africa (The Gambia, Kenya, Mali, and Mozambique) and 3 sites in Asia (Bangladesh, India, and Pakistan) [38]. The majority of subjects with MSD was aged 0 to 24 months. *Cryptosporidium* was the second most common cause of MSD in the first year of life (behind rotavirus) and the third most common cause in the second year of life (behind rotavirus and *Shigella*). A follow-on study in 2011 to 2012, GEMS-1A, studied less-severe diarrhea (LSD) at 6 sites in The Gambia, Mali, Mozambique, Bangladesh, India, and Pakistan [42]. Again, *Cryptosporidium* was identified to be a main pathogen—specifically, it was the third most common cause of LSD in the first year of life (behind rotavirus and enterotoxigenic *E*. *coli*) and the fourth most common cause of LSD in the second year of life (behind rotavirus, enterotoxigenic *E*. *coli*, and *H*. *pylori*). Extrapolating the results from GEMS-1 and GEMS-1A across sub-Saharan Africa and Asia predicted more than 7.5 million cases and 200,000 deaths attributable to cryptosporidiosis annually in those regions in children aged 0 to 24 months [29]. An analysis across all GEMS sites revealed *Cryptosporidium* to be an independent risk factor for mortality in toddlers aged 12 to 24 months, and a focused sub-analysis at the Mozambique site found *Cryptosporidium* to be 1 of only 2 pathogens (the other was enterotoxigenic *E*. *coli*) independently associated with increased risk of death [43,44]. It is worth noting that the methodology of GEMS involved limiting recruitment to the same number of children per week throughout the study period. Thus, seasonal variations in disease burden, which are likely relevant in cryptosporidiosis, may have been underestimated due to the study inclusion strategy.

MAL-ED was a birth cohort community surveillance study that took place between 2009 and 2014 and involved more than 2,000 children aged 0 to 24 months at urban and rural sites in Africa (South Africa, Tanzania), Asia (Bangladesh, India, Nepal, Pakistan), and South America (Brazil, Peru) [39]. Across all sites, *Cryptosporidium* was 1 of 4 pathogens most often associated with diarrhea in the first year of life (along with *Campylobacter*, norovirus, and rotavirus) and 1 of 6 pathogens most often linked with diarrhea in the second year of life (along with astrovirus, heat-labile enterotoxigenic *E*. *coli*, heat-stabile enterotoxigenic *E*. *coli*, norovirus, and *Shigella*). Heterogeneity in pathogen distributions was observed between study sites and the burden of *Cryptosporidium* varied. For example, *Cryptosporidium* was the most common pathogen associated with diarrhea at the Pakistan site in children aged 0 to 24 months.

The Global Burden of Diseases, Injuries, and Risk Factors Study 2015 (GBD 2015) from the Institute of Health Metrics and Evaluation (IHME) included a systematic analysis of diarrheal diseases, incorporating data from GEMS [4]. That analysis did not stratify by age in the first 5 years of life, and so the burden of disease in children aged 0 to 24 months specifically was not available. In all under-fives, *Cryptosporidium* was reported to be the second leading cause of diarrhea-related deaths (after rotavirus), responsible for an estimated 60,400 deaths.

### Children under 5 years of age with malnutrition

Diarrhea is a common comorbidity in children with malnutrition. Two prominent considerations that concern the interplay of cryptosporidiosis and malnutrition are the implications for children who are acutely malnourished (i.e., children with wasting; characterized by a low weight-for-age) and those with linear growth faltering (i.e., children that are stunted; characterized by a low height-for-age). Indeed, a “vicious cycle” is described between malnutrition and *Cryptosporidium* infection whereby chronic cryptosporidiosis is associated with linear growth impairment at the same time children with acute malnutrition may be predisposed to enteric *Cryptosporidium* infection [45,46].

In our own experience treating malnourished children in sub-Saharan Africa (SS, BA, PK), we have found that clinical management of acute watery diarrhea due to cryptosporidiosis is particularly difficult in children with wasting who have limited physiologic capacity to adapt to shifts in fluid and electrolyte balance. In these patients, it can be challenging to successfully rehydrate by oral routes, which is recommended when possible in most protocols. Using high volumes of oral rehydration solution specifically formulated for acutely malnourished children runs the risk of iatrogenic hyponatremia and cerebral edema [47,48], whereas parenteral fluid replacement in acutely malnourished children risks fluid overload and potentially fatal high-output heart failure [49]. These experiences at the bedside in caring for patients with cryptosporidiosis are reflected in observational studies that suggest an elevated risk of death in acutely malnourished children that are infected with *Cryptosporidium*. At a malnutrition ward in Zambia, 35% of children with cryptosporidiosis died compared to 14% without *Cryptosporidium* infection [49]. In Chad, the mortality rate in children with severe acute malnutrition and cryptosporidiosis was nearly twice that in children with malnutrition alone [50]. Similar results were reported in Uganda and, through modeling, a recent GEMS sub-analysis showed a trend toward association of cryptosporidiosis with additional risk of death in children with acute malnutrition [51,52].

The clinical impact of cryptosporidiosis in acutely malnourished children is concerning because the prevalence of infection in that population appears to be significant. In Bangladesh, the incidence of *Cryptosporidium*-induced diarrhea in malnourished children was 12.2 episodes per 100 child years compared to 7.3 in children without malnutrition (risk ratio 1.7; 95% confidence interval 1.1 to 2.6) [53]. *Cryptosporidium* was detected by PCR in approximately 20% of over 300 children with severe acute malnutrition in Malawi and Kenya (a comparison with the infection rate in well-nourished children was not reported) [54]. At sites in Ghana and India, approximately 10% of children with acute malnutrition were infected with *Cryptosporidium* as detected by PCR, microscopy, and ELISA, compared to 5% (Ghana) and 7% (India) in well-nourished children, though these differences were not statistically significant [55,56]. Other studies also reported a higher, albeit nonsignificant, incidence of cryptosporidiosis in malnourished children compared to those without malnutrition [52,57,58].

Stunting is a major global health problem affecting a staggering 23% of children in LMICs, which is largely attributed to a confluence of factors that includes nutrition deficiencies and enteric infection in impoverished environments [59,60]. Numerous cross-sectional studies, as well as a meta-analysis conducted as part of the Global Burden of Disease Study, associate *Cryptosporidium* infection with stunting [1,61]. Moreover, prospective investigations on 3 continents (Africa, Asia, and South America) have shown infection with *Cryptosporidium* in infancy and young childhood to be causal in stunting [41,62–65]. This relationship was additionally demonstrated through sub-analyses of longitudinal data collected through MAL-ED, which showed linear growth faltering at 2 of 8 sites (in India and Bangladesh) as well as a reduction in length-for-age at 2 years across 7 sites through a longitudinal modeling exercise [66,67].

Both asymptomatic (i.e., subclinical; without diarrhea) and symptomatic (i.e., associated with diarrhea) infections have been linked with stunting. In symptomatic infections, the risk appears to increase commensurate with increasing numbers of diarrheal episodes [62,63,65]. The long-term complications of *Cryptosporidium* infection at an early age may not be limited to growth faltering—impairments in general physical fitness and cognition later in childhood have also been recorded [68,69]. To date, the findings that demonstrate a potential role of cryptosporidiosis with impairments in growth and development have not been translated into clinical interventions; no studies have yet been conducted to assess the impact of treating enteric *Cryptosporidium* in young childhood to reduce the prevalence of stunting or other chronic sequalae.

### Immunocompromised children and adults

The increased morbidity and mortality imposed by *Cryptosporidium* [70,71] has important implications for the estimated 36 million PLWHA, more than 30 million of whom reside in LMICs [72,73]. Historically, the importance of *Cryptosporidium* as a human pathogen originated in the early 1980s as an opportunistic infection, particularly among PLWHA [8]. Cryptosporidiosis remains an advanced HIV/AIDS clinical staging criterion [74]. *Cryptosporidium* is among the most common intestinal parasites identified among children and adults living with HIV, and in many studies is the leading enteric parasitosis in those populations. Cryptosporidiosis may also be complicated by extraintestinal disease in immunocompromised patients (e.g., biliary tract and pulmonary infection) and, although they are rare, these complications are frequently fatal.

Recent investigations report that *Cryptosporidium* infection ranges between 5% to 50% of PLWHA in Africa, Asia, and the Americas [75–79]. A systematic review involving more than 100 studies estimated the overall prevalence of *Cryptosporidium* in this population to be 14% [80]. Absence of antiretroviral therapy and low CD4+ counts are known risk factors for increased incidence of cryptosporidiosis in PLWHA [81,82].

*Cryptosporidium* infection has also been reported as a leading cause of diarrhea in solid organ transplant (SOT) recipients receiving immunosuppressive therapies [83]. *Cryptosporidium*-induced diarrhea has been reported to occur in up to 30% of SOT recipients and is characterized by prolonged severe diarrhea, fluid and electrolyte depletion, and organ failure if untreated [83]. In a cohort of SOT patients followed in France, the incidence of cryptosporidiosis was highest in the first 6 months after transplantation [84]. Investigators recommended systematic screening for cryptosporidiosis prior to grafting and ensuring the consumption of *Cryptosporidium*-free water to patients when they are in highly immunocompromised states [84].

### Immunocompetent individuals affected by outbreaks

Outbreaks of cryptosporidiosis occur regularly worldwide with the largest recorded event in history having taken place in Wisconsin, United States, in 1993 (an estimated 400,000 affected) [85,86]. More than 600 outbreaks were reported in the US, England, and Wales from 2009 to 2017 [87,88]. Transmission in this context occurs mostly from recreational water exposure, animal contact, person-to-person exposure (e.g., in childcare settings), and foodborne routes. In the US, the number of reported cryptosporidiosis outbreaks has increased more than 10% annually in the past decade [87]. Better detection as a result of new diagnostic capabilities may be one of the reasons for the recent increases in incidence. Nonetheless, reporting of outbreaks, along with the numbers of individuals affected in any given outbreak, is generally considered an underestimate due to surveillance and case-finding constraints [87]. Taking those limitations into account, one analysis estimated more than a million symptomatic cases each year in just 3 European countries [89]. Underestimation of outbreaks is likely more pronounced in LMICs because of weak health information systems and the relative inaccessibility of diagnostics.

*Cryptosporidium*-specific therapy could potentially address acute disease in outbreak situations and prevent chronic complications. While cryptosporidiosis in immunocompetent individuals is usually self-limiting, it is more likely to persist compared with other enteric pathogens and in a minority of cases leads to hospitalization or even death [87,90]. In a Swedish outbreak beginning in 2010 that affected approximately 50,000 people, 35% of adult patients missed work for an average of 4 days to care for either themselves or their children [91]. As regards potential chronic complications, long-term follow-up of patients indicates the possibility of gastrointestinal or joint systems persisting at 1 or 2 years after acute infection; also, some patients had later been diagnosed with irritable bowel syndrome [92–95]. It is not clear whether the pathophysiology of chronic symptoms results from persistent or recurrent *Cryptosporidium* infection or true postinfectious sequelae (e.g., immunologically mediated). Future studies are needed to better understand the incidence and nature of chronic sequelae and whether timely and adequate treatment of acute infection may be preventative.

## Current treatment for cryptosporidiosis: Nitazoxanide

Nitazoxanide, approved by the US Food and Drug Administration in 2002, remains the only licensed medicine for treating cryptosporidiosis [102,103]. It was initially approved for use in children aged 1 to 11 years of age, and in 2004 was also licensed for older children and adults [103]. Nitazoxanide is a synthetic antiparasitic agent with broad in vitro parasitistatic activity against a variety of protozoa and helminths [102]. It is administered orally, heat stable, and generally well tolerated with reports of mild gastrointestinal side effects and occasional yellow discoloration of sclerae (which can, incidentally, cause alarm in patients who mistake that sign for jaundice) [104].

In protozoa, nitazoxanide inhibits the anaerobic energy metabolism enzyme pyruvate-ferredoxin oxidoreductase, though it is suspected that the drug’s mechanism of action (MOA) may also include other pathways [102]. The dependence of the efficacy of nitazoxanide on immune status suggests that its MOA may require a contribution from host immunity. Nitazoxanide amplifies host cell antiviral responses, notably interferons [105,106]. If the effect of nitazoxanide is mediated largely through interferons, it would be expected to be less efficacious when Th1 immunity is dysfunctional, as CD4 and NK cell function is required for parasite clearance [107], which could be a factor in nitazoxanide’s poor efficacy in HIV infection [11].

Several studies have shown nitazoxanide to significantly improve clinical response and to reduce the duration of diarrhea and oocyst shedding in immunocompetent adults and children with cryptosporidiosis [108,109]. Two randomized, placebo-controlled trials informed the regulatory approval of nitazoxanide and showed clinical response rates between 56% and 88% in immunocompetent adults and children compared to a placebo effect of 23% to 44% [10,108]. In these studies, parasitological clearance occurred in 52% to 75% of patients treated with nitazoxanide compared to 14% to 24% in patients treated with placebo [10,108].

In contrast to its effectiveness in immunocompetent individuals, the activity of nitazoxanide against cryptosporidiosis appears to be poor in acutely malnourished children, although data are limited. The most commonly cited study took place nearly 20 years ago in Zambia in which 25 HIV–negative, malnourished children with cryptosporidiosis were treated with a 3-day course of nitazoxanide; only 56% experienced resolution of diarrhea and 52% demonstrated oocyst clearance [10]. It has been theorized that the weakened immune system in children with malnutrition contributes to their inability to respond to nitazoxanide therapy [110].

In HIV–positive patients, nitazoxanide appears to lack efficacy altogether. A systematic review conducted in 2005 yielded 2 placebo-controlled trials that examined the efficacy of nitazoxanide in PLWHA in Mexico and Zambia. A meta-analysis of these studies showed that nitazoxanide failed to significantly reduce the duration and frequency of *Cryptosporidium*-associated diarrhea in immunocompromised patients and did not significantly affect oocyst clearance [99]. These findings are consistent with subsequent placebo-controlled trials in Egypt and Zambia [11,111].

In summary, evidence suggests that, with regard to treatment of cryptosporidiosis, use of nitazoxanide is limited in 3 areas in particular: It is not approved in infants under age 1 year, it is poorly effective in malnourished children, and it is ineffective in patients with HIV. There may be the opportunity to explore the use of nitazoxanide in combination with new anti-*Cryptosporidium* therapeutics should they become available.

## Treatment settings: Practical considerations

### Treatment settings

Diarrhea-related mortality in children under age 5 is highest in LMICs, and therefore, it is particularly urgent to assure safe and effective treatment options in those regions for patient groups that are known to be at high risk (Table 2). Disease-specific therapy would ideally be available wherever young children with diarrhea receive medical care, including at primary-, secondary-, and tertiary-level facilities. There are also clear needs for reliable access to anti-*Cryptosporidium* therapies at nutritional rehabilitation and HIV treatment centers. As the prevalence of *Cryptosporidium* infection in children is reported to be higher during the rainy seasons, greater medicine stocks would presumably be needed during those periods [112].

### Diagnostic considerations

Ideally, pathogen-specific treatment would be delivered based on the definitive diagnosis of cryptosporidiosis and reasonable certainty that the enteric presence of *Cryptosporidium* is causative to signs and symptoms of disease. Unfortunately, at this time, there are practical challenges to diagnosing cryptosporidiosis in the low-resource settings where it is most prevalent. Microscopy of fecal specimens is relatively inexpensive but relies on technical laboratory skills and consumables that are unlikely to be routinely available at resource-constrained and/or peripheral-level health facilities [113]. Immunological assays exist but are more expensive and complex to perform. PCR assays have been used frequently for research purposes in LMICs and have high sensitivity and specificity but are also expected to be prohibitively expensive for routine bedside use in resource-limited settings [114]. The clinical interpretability of PCR for diagnosis is also complicated by mixed infections where multiple organisms are present even if some are commensal or not responsible for active disease, a common scenario affecting up to 40% of children with diarrhea [50,115].

Antigen detection-based tests may be the most feasible approach to diagnosing cryptosporidiosis as part of routine clinical practice in low-resource settings. These assays can be used without sophisticated laboratories, require minimal training for health workers, and provide rapid results (often within 30 minutes of obtaining a stool sample). Rapid diagnostic tests (RDTs) are generally straightforward to deploy at the bedside in developing countries, as evidenced by their widespread use for diagnosis of malaria (more than 300 million RDTs for *Plasmodium falciparum* are used annually) [116]. There are several commercial RDTs for *Cryptosporidium*, and it has been demonstrated they can be used successfully in endemic regions in Africa and Asia [112,117]. However, though less expensive than immunoassays or PCR, the cost would likely still need to be substantially reduced to facilitate wide scale use in LMICs. Costs vary by manufacturer, though one program in Chad reported the cost of RDTs for *Cryptosporidium* to be approximately USD$10 per test [112]. For comparison, malaria RDTs are free when used in public health facilities in many countries, or otherwise often cost less than USD$1 [118].

### Empiric therapy and mass drug administration

There is a lack of evidence addressing the role of empiric therapy (i.e., treatment of acute diarrheal illness without confirmation of cryptosporidiosis diagnosis) or mass drug administration (MDA; i.e., therapies administered at population scale to asymptomatic individuals at high risk of infection) for cryptosporidiosis. However, scenarios are plausible where these methods could be warranted assuming a safe and effective medicine is available at an affordable cost. For example, in settings where the prevalence of symptomatic cryptosporidiosis is high and diagnostic tools are poorly available, presumptive treatment with an anti-*Cryptosporidium* therapeutic could be envisioned for patients at high risk of severe outcomes. The efficiency of this approach would presumably be maximized by precisely defining, to the extent possible, patient groups most likely to be infected. That could be determined, for example, according to age (e.g., infants), symptoms (e.g., prolonged or persistent watery diarrhea), time of year (e.g., wet season), or HIV or nutritional status. One study in progress, involving an Aboriginal population in Australia, is testing the empiric treatment of acute gastroenteritis in children using nitazoxanide under the hypothesis that children with a variety of enteric pathogens, including *Cryptosporidium*, would be safely treated and complications could be avoided [119]. Accumulated data from this study and similar investigations would be needed to provide sufficient justification to incorporate presumptive therapy into algorithms of care in standard treatment guidelines.

The population-wide benefits of periodic MDA are well recognized for addressing diseases such as trachoma, soil-transmitted helminthiasis, and other diseases [120–123]. For malaria, seasonal chemoprevention is a WHO-recommended practice in areas of high seasonal malaria transmission that has been implemented in more than 10 Sahelian countries, targeting over 10 million children with sequential monthly courses of presumptive antimalarial treatment [124,125]. The lessons to be learned from malaria seasonal chemoprevention programs could potentially help to inform MDA interventions to control cryptosporidiosis in the future, depending on progress with development of new anti-*Cryptosporidium* therapeutics. In cryptosporidiosis, rationale for MDA could stem from the extremely high rates of infection that have been observed in some patient groups (e.g., 97% of children in semi-urban slums in India were found to have acquired *Cryptosporidium* by age 3) [126], combined with emerging evidence, discussed above, concerning the potential role of cryptosporidiosis in growth faltering. Nitazoxanide has previously been proposed as an empiric treatment of presumed cryptosporidiosis and/or MDA, while acknowledging the research gaps that exist in efficacy and optimal dosing, cost, and potential development of drug resistance [127]. An ongoing study in Tanzania is assessing the impact of routine nitazoxanide administration (along with other antimicrobials) on linear growth in children [128]. As is the case with empiric therapy for acute disease, more data would be needed to inform the safety and utility of this practice with an ideal anti-*Cryptosporidium* therapeutic.

## The future of anti-*Cryptosporidium* therapeutics

### Ideal characteristics of an anti-*Cryptosporidium* therapeutic

An anti-*Cryptosporidium* therapeutic that is highly effective in the patient populations known to be at high risk of disease would constitute a critical addition to the limited interventions currently available for the investigation and treatment of diarrheal diseases. The ideal target product profile for a novel anti-*Cryptosporidium* agent has been previously described [129,130]. Briefly, the product should be safe and effective, particularly among populations most affected by the disease and thus licensed for use in children from 3 months of age onwards in addition to malnourished children and immunocompromised patients; be presented in a dosage form that is suitable for administration to young children; be available in a heat-stable formulation; be affordable; and have a simple dosage regimen to facilitate its use in low-resource settings [29,129,130]. In addition, it should be parasiticidal against both *C*. *hominis and C*. *parvum* leading to at least 95% reduction in oocyst shedding and resolution of diarrheal symptoms (i.e., “cure”) without any unmanageable safety risks [129].

### Anti-*Cryptosporidium* therapeutics in the pipeline

Despite its high burden and clinical impact, cryptosporidiosis remains an underappreciated global health concern, and antiparasitic treatment options are suboptimal. Several drug candidates (including the anticoccidial agent letrazuril, diclazuril, and interleukin-12) were tested in early phase clinical trials for the treatment of cryptosporidiosis in PLWHA in the early 2000s with limited success [131,132]. Similarly, the repurposing of a large number of medicines (including azithromycin, clofazimine, paromomycin, rifamycin, spiramycin, and HIV protease inhibitors) has been explored in the management of PLWHA affected by cryptosporidiosis, but as yet without sufficient clinical success [114,129,133–135]. To our knowledge, there has been no effort to identify parenteral anti-*Cryptosporidium* preparations that could be used to address extraintestinal disease.

In recent years, drug discovery research has focused on discovering novel compounds active against *Cryptosporidium* spp. Recent achievements in this area give cause for hope with diverse new chemical entities (NCEs) including *Cryptosporidium* lipid kinase PI(4)K inhibitors [136], bumped kinase inhibitors of CpCDPK1 [137], lysine tRNA synthetase (KRS) inhibitors [138], benzoxaboroles (e.g., AN7973) [139], a cleavage and polyadenylation specificity factor 3 (CPSF3) inhibitor [140], bicyclic azetidines phenylalanyl-tRNA synthetase inhibitors [141], a piperazine-based compound (MMV665917) [142], a choline-based phospholipid VB-201 [143], and other novel phenotypic screening hits [144]. Most of these NCEs have demonstrated desirable in vitro anti-*Cryptosporidium* activity and in vivo efficacy in immunocompromised mouse models in reducing fecal oocyst burden. Furthermore, several compounds, including BKI-1369 [145], KDU731 [136], MMV6659917, and AN7973 [139], have demonstrated resolution of diarrheal symptoms in neonatal calf models, a clinical model of cryptosporidiosis that closely resembles human infection. These promising preclinical candidates and other compounds described in the literature have largely demonstrated desired in vitro and in vivo anti-*Cryptosporidium* activity as defined by the target product profile [129,130]. However, further in vivo safety and pharmacological characterization are warranted, and none of these compounds have yet entered human trials.

## Conclusion

Emerging data is bringing the true burden of disease imposed by cryptosporidiosis into sharper focus. There is imperative now to ensure the successful translation of evidence and experience into actionable guidance for health workers to improve outcomes for patients. The only approved therapy for cryptosporidiosis appears to have important limitations for use in the 3 patient populations in LMICs for which an effective anti-*Cryptosporidium* therapeutic is urgently needed: young children aged 0 to 2 years, malnourished children aged 0 to 5 years, and PLWHA. Crisply defining the challenges to adequately detecting and treating cryptosporidiosis in these patient populations will be necessary to pragmatically overcome current clinical management barriers. At the same time, helping to educate global stakeholders about the newly appreciated burden of cryptosporidiosis will presumably aid advocacy efforts to support drug and vaccine discovery. Bolstered by recent accomplishments in early drug discovery, an urgently needed drug against pediatric cryptosporidiosis is a compelling vision in the near future through concerted collaborative efforts among clinicians, researchers, industry, and funding agencies in global health. Ideally, a virtuous cycle will be set in motion whereby the introduction of novel therapeutics would serve immediate clinical needs while helping to raise public health awareness about cryptosporidiosis and spur further innovation for disease management in areas of diagnostics, drugs, vaccines, and national disease prevention and control programs.
